# G0/G1 arrest and apoptosis induced by SARS-CoV 3b protein in transfected cells

**DOI:** 10.1186/1743-422X-2-66

**Published:** 2005-08-17

**Authors:** Xiaoling Yuan, Yajun Shan, Zhenhu Zhao, Jiapei Chen, Yuwen Cong

**Affiliations:** 1 Department of Pathophysiology, Beijing Institute of Radiation Medicine, Beijing, 100850, China

## Abstract

Severe Acute Respiratory Syndrome coronavirus (SARS-CoV), cause of the life-threatening atypical pneumonia, infects many organs, such as lung, liver and immune organ, and induces parenchyma cells apoptosis and necrosis. The genome of SARS-CoV, not closely related to any of the previously characterized coronavirus, encodes replicase and four major structural proteins and a number of non-structural proteins. Published studies suggest that some non-structural proteins may play important roles in the replication, virulence and pathogenesis of viruses. Among the potential SARS-CoV non-structural proteins, 3b protein (ORF4) is predicted encoding 154 amino acids, lacking significant similarities to any known proteins. Till now, there is no report about the function of 3b protein. In this study, 3b gene was linked with the EGFP tag at the C- terminus. Through cell cycle analysis, it was found that over-expression of 3b-EGFP protein in Vero, 293 and COS-7 cells could induce cell cycle arrest at G0/G1 phase, and that especially in COS-7 cells, expression of 3b-EGFP was able to induce the increase of sub-G1 phase from 24 h after transfection, which was most obvious at 48 h. The apoptosis induction of 3b fusion protein in COS-7 cells was further confirmed by double cell labeling with 7-AAD and Annexin V, the function of 3b protein inducing cell G0/G1 arrest and apoptosis may provide a new insight for further study on the mechanism of SARS pathogenesis.

## 

The outbreak of Severe Acute Respiratory Syndrome (SARS) posed a great global threat. SARS is a system disease which impairs many organs, such as lung, liver and immune organ. Respiratory distress and decreased immune function are the main causes of SARS patient death [1-3]. SARS was found to be caused by a novel coronavirus which was designated as SARS coronavirus (SARS-CoV), and the genome of SARS-CoV contains 11 to 14 open reading frames (ORF) and 5 to 8 potential non-structural proteins [4,5]. The virus non-structural proteins, which vary widely among different coronavirus species, are dispensable for virus replication. It has been known that some non-structural proteins play important roles in virulence and pathogenesis, such as X protein of hepatitis B virus and ORF 8 protein of bovine herpes virus 1U(S) [6,7].

SARS-CoV 3b (ORF4) (ZJ01, AY297028) encodes a 154-amino-acid protein, lacking significant similarities to any previously known proteins [8]. With bioinformatics analysis, using the PSORT II server, it was shown that C- or N-terminal signal peptide, coiled-coil regions and trans-membrane region allocation were not detected, however, two potential nuclear localization signals (NLS) were predicted. The cellular localization of 3b protein by confocal microscopy was performed and the nucleolus localization was confirmed. And the nucleolus localization signal sequences of 3b protein may localize in the C-terminal regions from 134 to 154 amino acids [9].

Nucleolus localization of SARS-CoV 3b protein suggested that the expression of 3b protein may interfere with cell cycle regulation in transfected cells. Flow cytometry was performed on COS-7 cells transfected with pEGFP-N1 (Clontech) or p3b/EGFP-N1, which allowed examination of two intra-culture populations with EGFP expression indicative of transfected cells. PI staining revealed that a similar pattern of phase distribution in EGFP positive and negative cells transfected with pEGFP-N1 from 24 h to 72 h periods (data not shown). However, for p3b/EGFP-N1 transfected cells, the phase distribution of positive cells was significantly different from that of negative cells (Figure 1). When compared with EGFP negative cells, the populations of positive transfected cells displayed a significant increase in G0/G1 phase, an obvious decrease in S phase, and an emergence of sub-G1 phase compared with negative cells at 24 h after transfection. At 36 h, the increase in G0/G1 phase in positive cells was also significant, but less than that at 24 h, while cells in S phase was more decreased, and cells in sub-G1 phase continued increase compared with negative ones. At 48 h, the increase in G0/G1 phase and decrease in S phase in positive cells were not significant, but the percentage of sub-G1 phase was significantly raised in positive cells (49.89% vs. 5.26%). From 60 to 72 h post-transfection, the increase in sub-G1 phase in positive cells was also significant, but less than that at 48 h, while G0/G1 phases in positive and negative cells were comparable (Figure 2). These data indicated that expression of 3b protein blocked or delayed the progression of cells from G0/G1 phase into S phase, and induced cells toward apoptosis.

**Figure 1:**
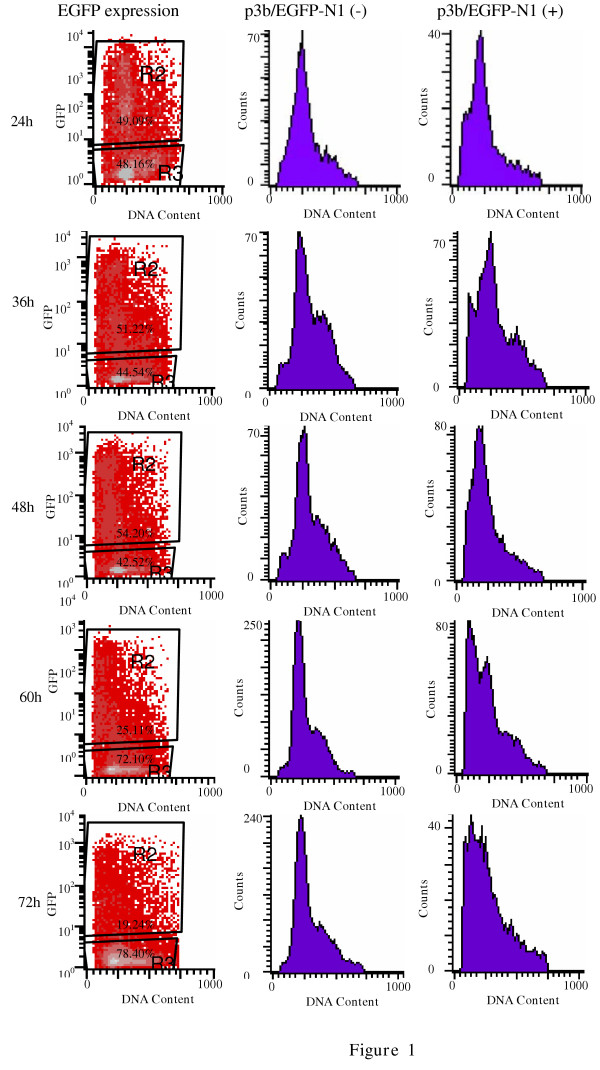
**Induction of cell cycle arrest and cell apoptosis by 3b protein expression**. p3b/EGFP-N1 plasmid was transfected into COS-7 cells, and the DNA contents of cells were measured by flow cytometry. EGFP expression positive and negative cells were gated with forward scatter (on the left row). The middle and right rows were the assay of cell cycle in p3b/EGFP-N1 negative and positive cells. In p3b/EGFP-N1 positive cells, sub-G1 phase was changed from 11.80% to 53.50%, 48.36% at 24 h or 36 h, 48 h separately. The proportion was decreased to 23.34 and 24.85% at 60 and 72 h respectively. However, in EGFP negative cells, the changes of sub-G1 phase were not obvious.

**Figure 2:**
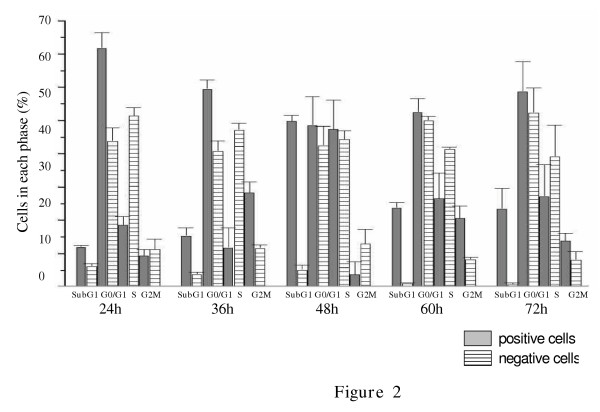
**Histogram of cell cycle arrest and cell apoptosis**. Histogram showing the percentages of cells at various phases of cell cycle. p3b/EGFP-N1 positive cells were showed with grey columns, and p3b/EGFP-N1 negative cells were showed with crisscross. Data were means of three independent experiment ± s.d. (bars).

Next, using similar method, we investigated the effects of p3b/EGFP-N1 on cell cycles in 293 and Vero cells. In these cells, the p3b/EGFP-N1 positive cells were arrested at G0/G1 phase compared with negative ones after transfection (55.25% vs. 37.17% in 293 cells and 82.27% vs. 56.00% in Vero cells at 48 h), but the percentages of sub-G1 phase in positive and negative cells were all too low and comparable. These data indicated that the role of 3b protein in inducing cell cycle G0/G1 phase arrest was a conserved character, but the apoptosis induction of 3b protein might be a cell type specific.

In order to further confirm the function of 3b protein in inducing cell apoptosis, a more definitive study using double cell labeling with Annexin V-PE and 7-AAD was performed. COS-7 cells were transiently transfected with pEGFP-N1 and p3b/EGFP-N1 separately. At 48 h after transfection, apoptosis analysis was carried out. As shown in figure 3, the different populations in EGFP positive and negative cells were measured by flow cytometry. It was revealed that the percentages in apoptosis and necrosis were both lower and comparable in the positive and negative pEGFP-N1 transfected cells (Figure 3b). However, for p3b/EGFP-N1 transfected cells, the apoptosis cells in the positive cells increased over 4-fold compared with that in the negative ones (Figure 3a). These data further demonstrated that over-expression of 3b protein could induce cell apoptosis.

**Figure 3:**
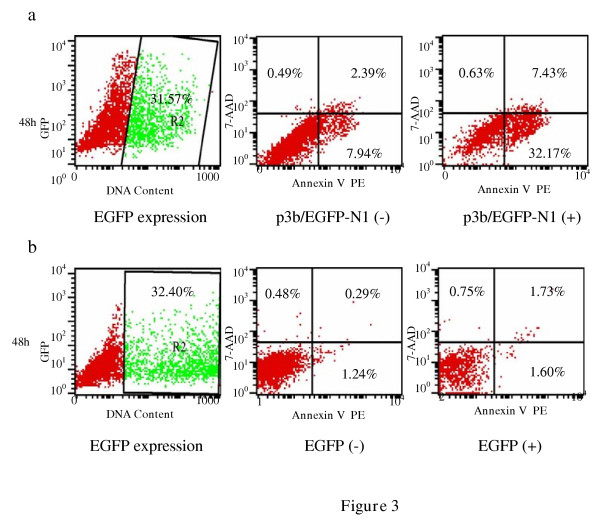
**Apoptosis assay of COS-7 cells transfected with p3b/EGFP-N1**. COS-7 cells were transfected with pEGFP-N1 and p3b/EGFP-N1 respectively. At 48 h after transfection, cells were collected and resuspended in binding buffer containing Annexin V-PE and 7-AAD, and then processed for flow cytometry analysis. On the left row, EGFP positive cells were gated. The middle and right rows were the results of EGFP negative and positive cells analyzed with Annexin V-PE and 7-AAD staining. In each box, the upper left corner included damaged cells, the lower left corner included viable cells, which were negative for 7-AAD and Annexin V-PE binding, the upper right corner included necrotic or late apoptotic cells, which were positive for Annexin V-PE staining and for 7-AAD uptake, while the lower right corner included apoptotic cells, which were Annexin V-PE positive but impermeable to 7-AAD. In p3b/EGFP-N1 transfected cells (a), the percentage of apoptosis cells in EGFP positive cells increased significantly, compared with negative ones, while there were no changes between positive and negative cells transfected with pEGFP-N1 (b). One of three experiments with similar results was shown.

Published data showed that massive necrosis was found in lung, spleen and lymph nodes in SARS patients. As compared with normal tissues, apoptosis cells increased significantly in the spleen, liver, lung, and lymph nodes of SARS patients. The apoptosis cells were further demonstrated to be pneumocytes, lymphocytes, and monocytes [10,11]. Taken together, the data we presented here, as well as the apoptosis and necrosis data of SARS patients, suggest that 3b is an apoptosis-related gene in SARS genome, which induce cell or tissue specific apoptosis in transfected cells.

## Competing interests

The author(s) declare that there are no competing interests.

## Authors' contributions

SY and ZZ conducted all the experiments. YX wrote the maunscript and coordinated the research efforts. CY conceived the study and edited the paper. CJ revised the article.
